# Comparison of One-Drill Protocol to Sequential Drilling In Vitro and In Vivo

**DOI:** 10.3390/bioengineering12010051

**Published:** 2025-01-09

**Authors:** Sihana Rugova, Marcus Abboud

**Affiliations:** 1 Department of Oral Biology and Pathology, Stony Brook University, Stony Brook, NY 11794, USA; 2 School of Engineering, Stony Brook University, Stony Brook, NY 11794, USA

**Keywords:** dental implant, straumann, BLT, SLActive, implant drills, implant bed preparation, heat generation, osteotomy, surgical drill bits, drilling protocols, osseointegration, bone-to-implant contact, thermal trauma

## Abstract

This study compares the heat generated during bone drilling using different protocols and implant systems, first in vitro and then in vivo with an animal model. In the experimental phase, thermal data were collected using an infrared camera while preparing implant beds in bone similes. The heat generated by a one-drill protocol with a new-generation drill bit and the Straumann BLT sequential drilling protocol was evaluated. The experimental study was then replicated in an animal model to assess the impact of these protocols on early osseointegration, measured by bone-to-implant contact (BIC) at three weeks post-surgery for Straumann BLT SLActive and Medentika Quattrocone implants. The results showed the BLT sequential protocol generated significantly more heat during drilling in bone similes compared to the new-generation drill bit. In the animal model, a histological analysis revealed a trend favoring shorter drilling protocols, with reduced drilling times and a potential advantage for osseointegration, though the BIC differences were not statistically significant. These findings suggest that minimizing the number of drilling steps and thermal stress may enhance osseointegration more effectively than advanced implant surface treatments. This aligns with emerging views on the importance of optimized drilling protocols and designs to reduce heat generation and better preserve surrounding bone structure.

## 1. Introduction

Dental implant treatment is a common option for patients with current or impending tooth loss, with about 2.5–5 million implants placed per year in the United States [1,2,3]. Implant success relies heavily on achieving optimal osseointegration, which begins with the preparation of a precise and stable implant bed [2,4,5,6,7,8]. Traditional sequential drilling protocols, while effective, often involve multiple steps that can increase the risk of procedural complexity, trauma, and prolonged surgical time [9,10]. Recent advancements in drill bit technology have aimed to address these challenges by simplifying the drilling process, minimizing invasiveness, and preserving bone health—all critical factors for creating a favorable healing environment and achieving high bone-to-implant contact.

Innovative approaches, such as the reduced or one-drill protocol using new-generation drill bits, are emerging as promising alternatives to conventional methods [11]. These advanced tools combine efficiency, ease of use, and positive clinical outcomes, offering potential benefits for both clinicians and patients. By reducing the number of drilling steps, these technologies aim to streamline procedures without compromising the biological prerequisites for osseointegration, ultimately contributing to the long-term stability of implants.

This study investigates the clinical and biological impacts of a simplified drilling protocol using a new-generation, advanced starter drill bit. An experimental study is first performed to assess heat produced during implant bed preparation in a standardized environment with standard bone similes, a drill press, and an infrared camera to capture thermal data during bone drilling. The same drilling protocols are then implemented in an animal study to evaluate the histological outcomes associated with different implant surface designs and drilling protocols over a three-week period. By comparing these variables, the research seeks to determine the influence of simplified drilling techniques on bone healing, implant stability, and overall procedural outcomes. The findings aim to provide insights into the feasibility of incorporating advanced drill bit technologies into routine clinical practice, potentially reshaping implant placement protocols toward a more efficient and patient-friendly approach. We hypothesize that a shorter drilling protocol will produce less thermal trauma and therefore higher bone-to-implant contact during the early stages of osseointegration.

## 2. Materials and Methods

**Figure 1:**
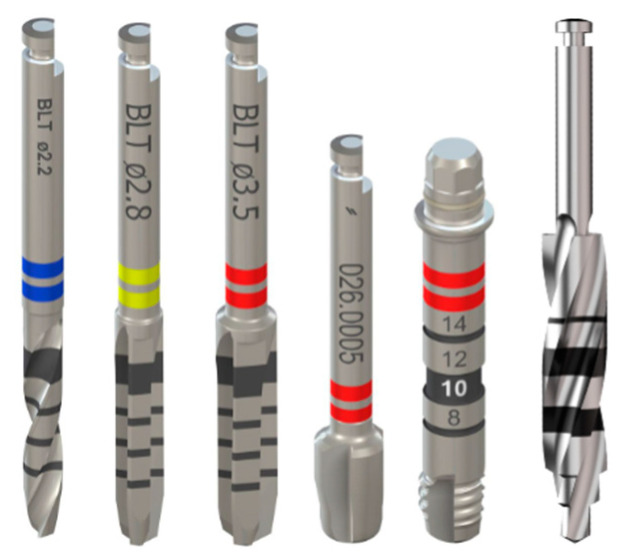
Images of the drill bits used in study. From left to right: ø 2.2 mm BLT, ø 2.8 mm BLT, ø 3.5 mm BLT, ø 4.1 mm profile drill, ø 4.1 mm BLT tap, and ø 4.0 mm new-generation drill bit.

Experimental study. The heat generated when preparing an implant bed with Straumann Bone Level Tapered (BLT) drill bits (Straumann, Basel, Switzerland) or with the ø 4.0 mm new-generation drill bit (Blitz GmbH, Munich, Germany) were assessed using the experimental protocol outlined by Rugova and Abboud (2024) [12]. Table 1 lists the two drilling groups tested and their corresponding spindle speeds. The thermal data gathered from the two groups were combined into a single graph presented in the results. All drilling tools studied are shown in Figure 1. The temperatures achieved during bone drilling were recorded and compared statistically by a Student’s *t*-test. Statistical significance was established as *p* < 0.05; *n* = 10.

**Figure 2:**
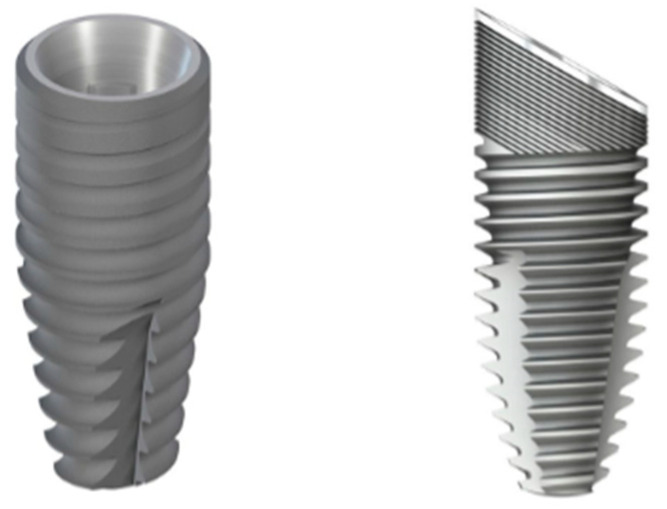
Image of the implants used in this study; ø 4.1 × 10 mm BLT (**left**), ø 4.1 × 10 mm Quattrocone (**right**).

Animal study. The same drill bits and drilling protocols were then implemented in an animal study in order to place Straumann BLT implants (Straumann, Basel, Switzerland), with the SLActive surface, which is a chemically modified, sandblasted, and acid-etched surface that is highly hydrophilic. In addition, Straumann implants, Quattrocone 30 (Medentika-Straumann Group Brand, Hügelsheim, Germany) with a standard sandblasted, and an acid-etched surface were used to evaluate the effect of the drilling protocol on osseointegration. The implants used are shown in Figure 2.

Five male sheep, each weighing approximately 120 pounds, were utilized in this study and randomly assigned to a study group. The species Ovis Aries, commonly known as domesticated sheep, were selected due to their similarity in size, weight, composition, metabolism, and bone remodeling to humans, making them a suitable experimental model [13,14,15,16]. The animals were adjusted to their new environment at least 2 weeks prior to surgery. Twelve implants were surgically inserted in the mandibles of each sheep, six in the left jaw and six in the right, for a total of sixty implants across all animals [2,17,18,19,20,21]. Twenty implants were placed per group to ensure a statistically relevant sample size. The minimum number of implants for statistical relevance is 15; thus, if any implants are lost in this study, the necessary sample size can still be achieved. This animal study was reviewed and approved by the veterinary staff of the experimental animal facility as well as the Institutional Animal Care and Use Committee (No.16ABBOUD002). Pre-anesthetic preparations, general anesthesia, shaving, and antisepsis procedures were handled by the staff veterinarian of the experimental facility, similar to other studies [15,22,23,24].

General anesthesia: All surgical procedures were completed under general anesthesia to mitigate the animals’ pain and anxiety. Prior to anesthesia, the sheep were deprived of solid food for 24 h and liquids for 6 h to reduce the risk of regurgitation and aspiration of stomach contents, which can lead to suffocation and pneumonia. The veterinarian premedicated the animals with enrofloxacin (2–2.5 mg/kg i.m., prophylactic antibiotic) 48 h preoperatively, and ketamine (5–10 mg/kg i.m., anesthetic agent), midazolam (0.1–10 mg/kg i.m., benzodiazepine with anxiolytic properties), and torbugesic (2 mg/kg i.m., opioid analgesic with sedative properties) 20–30 min prior to the start of the surgical procedure to relax vagal tone. After confirming the absence of pain reflexes, cephalic vein cannulation was performed, and Ringer’s solution was connected to maintain animal hydration and electrolyte balance. Orotracheal intubation with cuff inflation was preformed, and 2–5% isoflurane by inhalation was administered to maintain general anesthesia. Glycopyrolate (0.01–0.02 mg/kg i.m.) was provided to reduce the hypersalivation often seen in ruminants under anesthesia. Localized administration of 2% lidocaine with 1:100 k epinephrine was utilized for intraoperative analgesia. General anesthesia was monitored using various equipment measuring heart rate, respiratory rate, oxygen saturation, end tidal oxygenation, body temperature, and ocular/pedal reflexes. Body temperature was maintained with a warm blanket.

**Figure 3:**
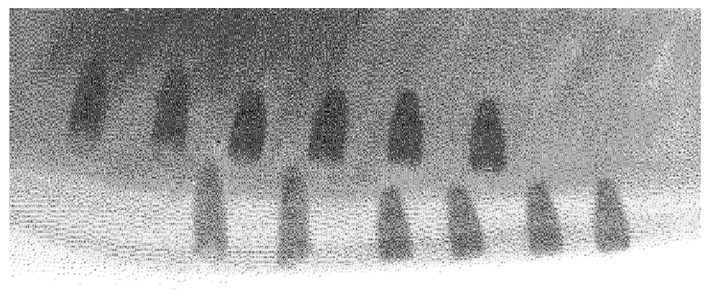
Radiograph showing placement of implants after surgery.

Surgical procedure: The surgical sites, the right and left lateral mandible, were shaved and cleaned with antiseptic iodine solution. The animals were draped with sterilized surgical cloths to isolate and define the surgical regions of interest. Surgery was performed by a single oral surgeon to maintain surgical consistency. An incision of approximately 10 cm in length along the long access of the mandible was made with scalpel size #3. Once the bone was exposed, the implant sites were marked with a round burr so that each implant would be 3 mm away from each other in their final positions. The implants and surgical protocols for each implant are listed in the tables below. All drilling was performed with sterile irrigation via a surgical handpiece throughout the drilling procedures. Insertion torques were measured with the surgical implant motor during implant insertion into the mandible. Following each surgical protocol, the implants were placed 1–2 mm subcrestal, the healing caps screwed into the implants, primary soft tissue closure achieved with resorbable 4-0 vicryl sutures placed continuously on the superficial layer of tissue, and interrupted sutures placed in internal layers of tissue. Radiograph of the implant placement are shown in Figure 3.

Implants and Implant Bed Preparation Groups (Table 2):

- Control group—The manufacturer protocol was followed according to the IFU. Drilling protocol was completed in sequence (ø 2.2 mm → ø 2.8 mm → ø 3.5 mm → ø 4.1 mm profile drill bit), followed by a bone tap device that reproduces the implant thread design in the bone, and placement of the BLT implant.
- Group A—The manufacturer’s drilling protocol was modified by replacing their first three drill bits (ø 2.2 mm, ø 2.8 mm, ø 3.5 mm) with a new-generation ø 4.0 mm advanced starter drill bit, which generates reduced heat during drilling and minimizes the need for incremental drilling steps [11]. This was followed by a bone tap device to reproduce the implant thread design in the bone and finally placement of the BLT SLActive implant.
- Group B—A one-drill protocol was used, followed by the placement of the Quattrocone 30 implant (ø 4.0 mm new-generation starter drill bit → ø 4.1 mm implant). It should be noted that the advanced started drill bit does not fully correspond to the form and shape of the implant used in this study.

**Table 2:** Animal study groups.

Study Groups	Surgical Protocol	Spindle Speed (rpm)	Insertion Torque
Control group. BLT protocol with BLT implant	ø 2.2 mm drill bit	800	
	ø 2.8 mm drill bit	600	
	ø 3.5 mm drill bit	500	
	ø 4.1 mm profile drill bit	300	
	ø 4.1 mm bone tap	15	
	ø 4.1 × 10 mm BLT implant placed	15	35–45 Ncm
Group A. Modified protocol with BLT implant	ø 4.0 mm new-generation drill bit	1000	
	ø 4.1 mm profile drill bit	300	
	ø 4.1 mm bone tap	15	
	ø 4.1 × 10 mm BLT implant placed	15	35–45 Ncm
Group B. New-generation drill bit with Quattrocone 30 implant	ø 4.0 mm new-generation drill bit	1000	
	ø 4.3 × 11 mm Quattrocone 30 implant placed	15	35–45 Ncm

Post-operative care: The animals were monitored daily and assessed on a pain score to determine the need for analgesics. A prophylactic antibiotic, enrofloxacin, was continued for 5 days post-operatively, and pain medication, banamine (0.5–2.2 mg/kg i.m.), was given twice a day for 3 days. Water was given ad libitum, and food and forage were provided twice a day. Wounds were evaluated daily for signs of infection.

Tissue collection and processing: At the end of the three-week experimental period, the animals were euthanized by veterinary staff via intravenous administration of pentobarbital sodium (100–150 mg/kg). Histological samples were then prepared and blindly analyzed by New York University Department of Biomaterials and Biomimetics (New York, NY, USA), as written by Donath et al. [25]. The specimens were kept in 10% buffered formalin solution for 24 h. Specimen blocks, each containing a single implant, were washed in distilled water and gradually dehydrated from 70% to 100% ethanol. Once dehydrated, the specimens were embedded in a methacrylate-based resin (Technovit 9100; Heraeus Kulzer GmbH, Hanau, Germany) and then cut into slices about 300 μm thick with a diamond saw (Isomet 2000; Buehler Ltd., Lake Bluff, IL, USA) along the long axis of the implant through its center, glued onto acrylic plates with acrylic adhesive (Aron Alpha Industrial Krazy Glue; Elmer’s Products, Inc., Columbus, OH, USA), and allowed to cure for 24 h. Processing was then continued with sequential grinding and polishing using abrasive papers with increasing grit size (400, 600, 800, 1200, and 2400; Buehler Ltd.), coated with silicon carbide, and irrigated on a grinding machine until sections achieved a final thickness of about 30 μm. The sections were then stained with Stevenel’s Blue and Van Giesons’s Picro Fuschin (SVG) stains and scanned with an automated slide scanning system and specialized computer software (Aperio Technologies, Vista, CA, USA). For histomorphometric evaluation, an imaging analysis software (ImageJ, NIH, Bethesda, MD, USA) was used to quantify and evaluate osseointegration parameters around the peri-implant surface: bone-to-implant contact (BIC) [24,25,26,27]. Statistical evaluation of BIC was performed using the Kruskal–Wallis test. Statistical significance was set at 95%, and post hoc testing for multiple comparisons used the Dunn test. The analysis was randomized with the group labels being anonymized so that the analysts were unaware of which labels corresponded to the treatment or control group.

## 3. Results

**Figure 4:**
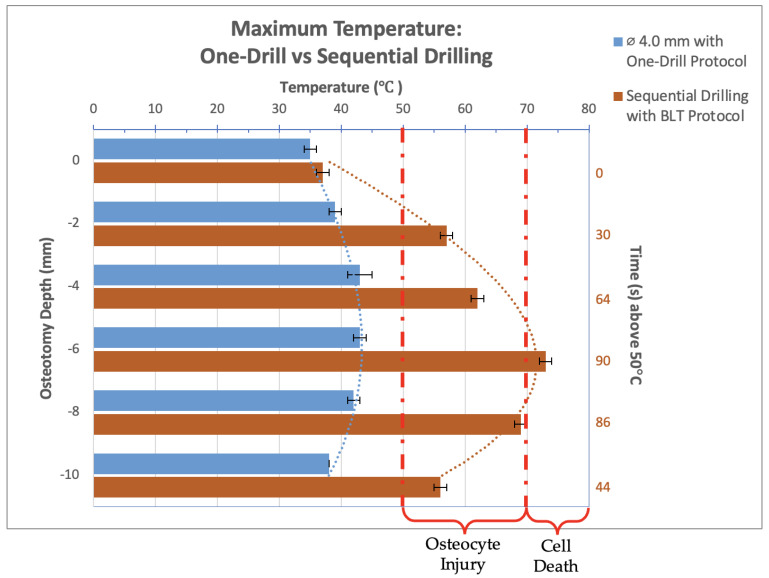
Bar graph showing maximum temperatures reached when drilling into bone to a depth of 10 mm with the 4.0 mm diameter drill bit used in a one-drill protocol compared to the Straumann BLT drill bits used in series. The temperature range of bone cell injury and cell death are marked on the graph. The left axis shows drilling depth (mm), while the top axis shows the maximum temperature reached (°C) at that depth. The right axis shows the duration (s) when temperatures exceeded 50 °C.

Experimental study. Thermal video recordings taken during the implant bed preparation procedure for the 4.0 mm diameter new-generation drill bit used in a one-drill protocol and Straumann BLT drill bits used in sequential drilling were analyzed and maximum temperatures at osteotomy depths of 0, 2, 4, 6, 8, and 10 mm were recorded and organized into the bar graph seen in Figure 4. The results for the ø 4.0 mm new-generation drill bit tested at three different spindle speeds were previously published; however, for this study, only the results at an rpm of 1000 are shown as this is the spindle speed used in the animal study presented [11]. The graph is marked with the label “osteocyte injury” to show where osteocyte damage is expected to varying degrees when 50 °C is exceeded. It is also marked with the label “cell death” to show where immediate cell death can be observed starting at 70 °C. The red numbers to the right of the graph show the time in which the osteotomy was observed to be above 50 °C. These numbers only reflect the times recorded for the sequential drilling protocol using the Straumann BLT drill bits as the 4.0 mm diameter new-generation drill bit did not exceed 50 °C and therefore did not have values to show.

The maximum temperature reached using the 4.0 mm diameter drill bit at a spindle speed of 1000 rpm never exceeded the temperature threshold for irreversible tissue damage, 50 °C for 30 s or 70 °C for 0 s. The highest temperature recorded was 43 °C, occurring at a depth of 4 and 6 mm. The temperatures recorded were within the range of 35–45 °C. The Straumann BLT drill bits used in sequential drilling reached or exceeded 50 °C for 30 s at all depths except 0 mm. At a depth of 2 mm, 50 °C was exceeded for 30 s. At depths of 4, 6, and 8 mm, 50 °C was exceeded for over one minute. At the 6 mm depth, 70 °C was exceeded. Sequential drilling with the BLT drill bits showed consistently excessive high temperatures, indicating irreversible tissue damage at most depths, with immediate cell death at the 6 mm depth.

**Figure 5:**
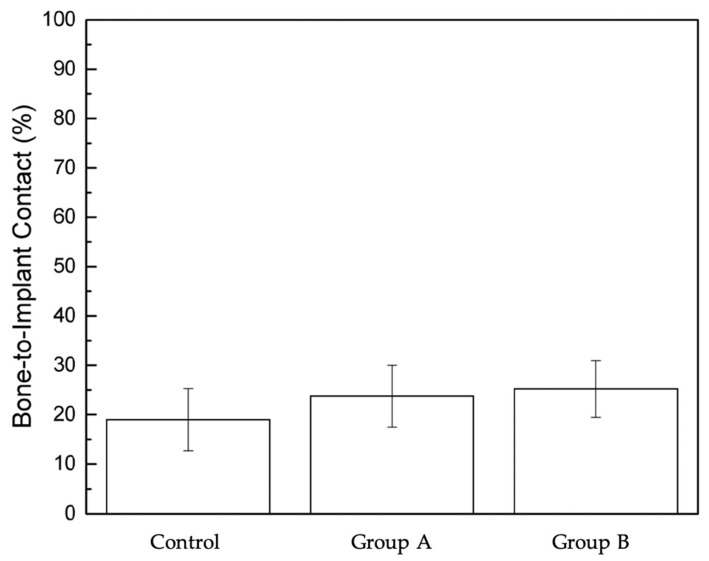
Bar graph showing bone-to-implant contact percentage at three weeks post-implant placement.

**Figure 6:**
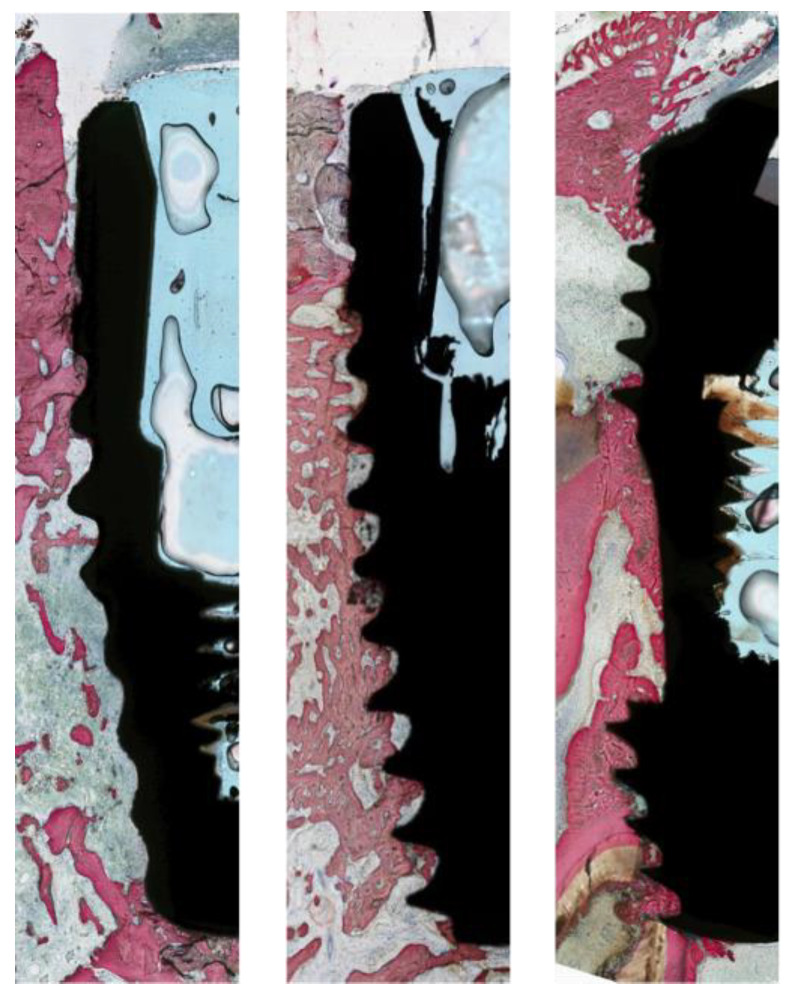
Histological section of implants at three weeks post-implant placement. From left to right: control group, Group A, Group B.

Animal study. The bone-to-implant contact results three weeks after surgical implant placement (Figure 5 and Figure 6) reveal a trend favoring the modified and single drilling protocols (Groups A and B) over the conventional approach; however, no statistically significant differences emerged between the three groups. The control group, which employed the Straumann BLT sequential protocol as recommended by Straumann, demonstrated slightly lower BIC values than Groups A and B at three weeks post-surgery. This group is the only one in the study using exclusively fully customized drill bits specifically designed to match the implants being tested. The use of tailored drill bits ensures that the implant bed closely mirrors the implant’s shape and size, optimizing BIC and enhancing primary stability. This precise alignment reduces micro-gaps and supports a more secure fit within the bone, setting a strong foundation for osseointegration.

Group A, which utilized a modified, shorter drilling protocol, achieved slightly higher BIC values than the control group while also showing lower data variation.

Group B, which implemented an even shorter, one-drill protocol, achieved results comparable to Group A, despite using a standard sandblasted and acid-etched implant surface.

Groups A and B showed significant clinical efficiency compared to the control group due to their reduced implant bed preparation drilling protocols. Group A averaged 4 min per procedure, while Group B averaged 2 min per procedure, compared to 12 min for the control group’s sequential approach.

## 4. Discussion

The results of the experimental study support the BIC trends measured at the early osseointegration stage in the animal study. While the animal study did not find statistically significant differences between the three groups concerning BIC values three weeks post-operatively, the observed trends reveal a favorable performance for the modified drilling protocols (Groups A and B) over the traditional, sequential, approach used in the control group. The recommended Straumann BLT manufacturer sequential protocol employed by the control group showed slightly lower BIC values compared to the modified protocols, suggesting that the standard method might not optimize bone-to-implant integration as effectively as the alternative approaches. This trend aligns with the evolving understanding of how drilling protocols and designs can influence osseointegration by reducing unnecessary steps, decreasing heat generation, and ensuring better preservation of the surrounding bone structure [28,29,30,31].

In Group A, a modified, shorter drilling protocol tailored for the Straumann BLT SLActive implants led to a slight increase in BIC values compared to the control group. Moreover, a lower variability in BIC outcomes was exhibited, indicating more consistency across the sample population. This reduced variability could signify a more predictable osseointegration process, which is particularly advantageous in settings where reliability and reproducibility of outcomes are paramount to clinical success. Notably, the only initial drill bit used in this protocol was the new-generation heat-reducing drill bit. The subsequent steps continued with the recommended manufacturer’s instruments. Previously performed experimental research has shown that standard drill bits generate excessive heat which can translate negatively on the native bone being operated on [32]. The mixture of drill types used in Group A may limit the protocol’s ability to optimize results, as later stages of drilling may still generate additional heat and contribute to bone trauma and limit timely heating, suggesting an area for further investigation. Additional studies should explore the outcomes of using exclusively new-generation drill bits throughout the protocol to determine whether this consistency can be further enhanced without reverting to standard drill products.

Group B took an even more streamlined approach with a one-drill protocol, achieving BIC values comparable to Group A despite relying only on a standard sandblasted and acid-etched implant surface compared to the patented SLActive surface marketed to customers for its ability to “enhance bone formation” and stimulate “faster osseointegration” [33,34]. The effectiveness of this one-drill approach is particularly significant, as it suggests that comparable levels of BIC can be achieved without the need for costly advanced implant surface treatments. This outcome highlights the potential of new-generation drill bits to not only reduce drilling time and steps but to also contribute actively to the critical bone–implant contact area. By minimizing drilling steps and the associated thermal stress on the bone, this protocol may support osseointegration more efficiently, ensuring healthy bone preservation and optimal implant integration [9,35,36]. The reduced treatment time would benefit patients directly by minimizing chair time and potential discomfort. The results suggest that the one-drill protocol is an effective and efficient alternative to traditional methods, supporting favorable osseointegration with added procedural simplicity. Further studies are needed to fully understand the significance of the drill bit in this process and to evaluate whether its influence on BIC might be as important as, or even more critical than, the properties of the implant itself.

The Group A and Group B protocols demonstrated promising trends just three weeks post-operatively toward enhanced BIC values and more consistent outcomes, potentially offering viable alternatives to the classic Straumann BLT sequential protocol. The lower variability in Group A and the resource efficiency in Group B highlight the value of these modified approaches for clinical applications. Further research may help to solidify and validate these trends and explore the clinical benefits of these protocols in larger patient populations.

An interesting implication of this finding is that the drill bit itself could play a role as important as, or potentially more critical than, the implant’s surface characteristics in achieving favorable surgical outcomes. The new-generation drill bits appear to facilitate both ease of procedure and quality of bone preparation, underscoring the need for further research into the drill bits’ impact on bone–implant contact. If future studies confirm this influence, the industry could see a shift toward prioritizing drill bit technology as a pivotal factor in implant success, potentially reshaping standard protocols around the specific attributes of the drill rather than focusing solely on implant design [37,38,39].

Another promising implication of the new-generation drill bits is their potential to enhance clinical outcomes for medically compromised patients. Patients with conditions such as osteoporosis, diabetes, or other systemic health issues often face increased risks during implant procedures, primarily due to compromised bone quality and delayed healing responses [40,41,42]. The advanced design of these drill bits, which generates less heat and preserves healthy surrounding bone, could be especially beneficial in such cases, where preserving bone vitality is crucial. By minimizing thermal trauma and reducing the number of drilling steps, these drill bits may support more effective osseointegration even in patients with suboptimal bone density or healing capacity. If validated through further research, these drill bits could become instrumental in adapting implant protocols to meet the specific needs of medically compromised patients, thereby broadening the scope of patients who can safely undergo successful, predictable implant procedures.

The time efficiency of the reduced implant bed preparation drilling protocols in Groups A and B shows a clinical benefit to both patients and providers. Procedures in these groups averaged only 2–4 min, a substantial reduction from the 12 min average of the control group’s sequential approach. This decrease in time is highly advantageous, especially in busy clinical environments, as it allows for more streamlined workflows and a reduction in the number of clinical steps and instruments needed. For clinicians, this translates to fewer procedural complexities, lower resource use, and ultimately, greater overall efficiency. Such time-saving protocols could be particularly useful in high-volume practices or in settings where patient turnover and operational efficiency are essential. Furthermore, the reduced treatment duration benefits patients as well, minimizing chair time, discomfort, and potential procedural anxiety.

It is important to note that the new-generation drill bits used in this study were not customized to match the form and shape of the specific implants being tested. Customizing the drill bit to align precisely with the implant’s geometry could further optimize implant bed preparation, potentially enhancing BIC and osseointegration. A drill bit specifically designed to mirror the implant’s dimensions would likely provide superior outcomes by ensuring an ideal fit, promoting closer contact between the implant and surrounding bone, and consequently increased primary stability. Based on this assumption, a one-drill protocol using a customized drill bit is anticipated to yield even better results, potentially achieving higher BIC values and more robust osseointegration. Future studies exploring the effects of such customization could provide valuable insights and may set a new standard in implantology, aligning drill bit and implant design for optimized clinical outcomes.

The one-drill protocol thus combines practical advantages with clinical effectiveness. Its ability to produce comparable BIC values to more elaborate protocols, while using fewer steps and reducing treatment time, points to an efficient alternative to traditional methods. Moreover, the streamlined process suggests a higher degree of standardization, which can lead to more consistent outcomes across different clinicians and clinical environments. This standardization is valuable not only for clinicians aiming to optimize treatment protocols but also for patients, who may benefit from quicker, less invasive procedures with predictable outcomes [43].

None of the new-generation drill bits used in this study were utilized to undersize the osteotomy with the intention of increasing implant stability. While certain drill bit designs intentionally undersize the osteotomy to create controlled bone compaction, thereby enhancing primary stability, this approach was not applied here. Controlled bone compaction can be advantageous as it compresses the surrounding bone, increasing initial BIC and potentially encouraging faster osseointegration. This compaction technique aims to provide a snug fit around the implant, promoting new bone growth in close proximity to the implant surface when working in lower bone densities [44,45,46].

However, optimal drill bits must strike a careful balance between sufficient compaction for stability and the preservation of open spaces within the bone for blood flow, nutrient delivery, and healing. Excessive compaction can inhibit the natural healing process, underscoring the importance of drill bit designs that offer controlled, moderate compaction without jeopardizing bone vitality [47,48,49]. Future research could explore the benefits of undersizing osteotomies using these advanced drill bits to assess their impact on implant stability, BIC, and overall osseointegration, particularly in clinical scenarios where enhanced primary stability is desired.

It is important to acknowledge the inherent limitations of using an animal model, as it cannot fully replicate the complexities of human physiology and clinical conditions. Animal studies provide valuable preliminary data but may not accurately reflect the outcomes observed in human patients. Sheep mandibular bone differs from human mandibular bone in several significant ways, primarily in terms of density, structure, and healing characteristics. Sheep mandibular bone tends to be denser and harder than human mandibular bone, causing differences in healing ability and surgical outcomes [50]. This increased density can make drilling more challenging and may not fully represent the conditions encountered in human patients, where bone density varies greatly depending on factors like age, health, and anatomical location. The sheep in this study exhibited significantly thicker cortical bone compared to what is often found in human mandibles with a less defined and smaller proportion of cancellous bone. Human mandibles typically have a more balanced distribution of cortical and cancellous bone, especially in areas where implants are placed. This difference affects bone remodeling and healing responses post-implantation. Due to these differences, while sheep models are useful for studying basic implantology concepts, they may not accurately predict implant performance or osseointegration outcomes in human mandibles. As such, data from sheep studies should be interpreted with caution and complemented by human clinical studies to obtain more realistic insights.

This animal study included the placement of sixty implants in five sheep; however, only fifty-four implants were placed, as halfway through surgery on one sheep, it started to show poor vitals. Thus, surgery was stopped to ensure the safety of the animal.

## 5. Conclusions

The findings in this study suggest that simplifying drilling protocols may provide a balance between procedural simplicity and favorable clinical outcomes, contributing to improved experiences for both clinicians and patients. Further research is warranted to validate these trends in larger, diverse patient populations and to explore the full clinical implications of using advanced drill bit technologies throughout the drilling protocol. The insights from these studies could shape future implant protocols, emphasizing a simplified, effective, and patient-friendly approach to implant placement.

## Figures and Tables

**Table 1:** Drilling groups tested in vitro.

Drilling Groups	Drill Bit Diameter (mm)	Spindle Speed (rpm)
One-drill protocol: new-generation drill bit	ø 4.0	1000
Sequential drilling: BLT protocol	ø 2.2	800
	ø 2.8	600
	ø 3.5	500
	ø 4.1	300

## Data Availability

The dataset is available on request from the authors.
